# Effects of exenatide on urinary albumin in overweight/obese patients with T2DM: a randomized clinical trial

**DOI:** 10.1038/s41598-021-99527-y

**Published:** 2021-10-08

**Authors:** Chao Kang, Qiao Qiao, Qiang Tong, Qian Bai, Chen Huang, Rong Fan, Hui Wang, Kanakaraju Kaliannan, Jian Wang, Jing Xu

**Affiliations:** 1 Department of Nutriology of The General Hospital of Western Theater Command, Chengdu, Sichuan China; 2 Department of Endocrinology of Xinqiao Hospital, Army Medical University, Chongqing, China; 3 Department of Nutriology of Xinqiao Hospital, Army Medical University, Chongqing, China; 4 Laboratory of Lipid Medicine and Technology, Department of Medicine, Massachusetts General Hospital and Harvard Medical School, Boston, USA

**Keywords:** Type 2 diabetes, Randomized controlled trials

## Abstract

In this study, we investigated the effect of exenatide (EXE), a glucagon-like peptide (GLP)-1 receptor agonist, on kidney function, obesity indices, and glucose control in overweight/obese patients with type 2 diabetes mellitus (T2DM). A total of 159 overweight/obese patients with T2DM were randomized to the EXE group or insulin glargine (GLAR) control group for a total treatment period of 24 weeks. EXE intervention significantly reduced the urine albumin concentration (UAC) at week 12 and 24 endpoints (*P* < 0.001 at week 12 and 24). The levels of the anthropometric, glucose and lipid parameters (TG and HDL-c), and inflammation biomarkers (CRP and TNF-α) in the EXE group were improved at 12 weeks or 24 weeks, respectively. Meanwhile, a comparison between two groups showed significant changes in anthropometric parameters, glucose parameters, lipid parameters (TG and HDL-c), and Inflammation biomarkers (CRP, IL-6, and TNF-α). Serum fibroblast growth factor 21 (FGF21) was increased in the EXE group (*P* = 0.005) at week 24, and the change was significantly improved compared with GLAR group (*P* = 0.003). Correlation network analysis showed that FGF21 had a more central role in improving metabolism in the EXE group, and the change of FGF 21 was significantly negatively correlated with UAC at week 12 and week 24, respectively (*r* = − 0.297, *P* = 0.010; *r* = − 0.294, *P* = 0.012). Our results showed that EXE could help patients improve UAC, glycemic levels, and inflammatory biomarkers after a follow-up period of 24 weeks intervention. These EXE effects may be partly mediated by FGF 21, indicating that EXE is an effective and safe way to control albuminuria in overweight/obese patients with T2DM.

## Introduction

Obesity, especially abdominal obesity, is closely associated with diabetic nephropathy (DN), a major cause of morbidity and a key determinant of mortality in patients with type 2 diabetes mellitus (T2DM)^1^. A previous study reported that about 25% of diabetic patients are affected by DN, and DN is the primary cause of end-stage renal disease (ESRD) in developed countries^2^. Early DN shows glomerular nodular lesions and hyaline arteriolar renal pathological changes, and urinary albumin excretion continues to increase, resulting in microalbuminuria. However, there is no glomerular filtration rate (GFR) decline but has entered the early DN stage. Therefore, microalbuminuria is a more reliable and sensitive early detection indicator for DN^3^.

Exenatide (EXE), a natural glucagon-like peptide 1 (GLP-1) analog, is a synthetic incretin-mimetic peptide. Numerous studies have demonstrated that EXE improves glycemic control and reduces body weight in patients with T2DM^4^. The DURATION-3 trial with 456 patients showed that once-weekly EXE treatment for 3 years significantly reduces HbA1c concentrations compared with insulin glargine^5^. Some evidence suggests that EXE may affect renal outcomes beyond glucose-lowering. Four weeks of EXE intervention could increase nitric oxide synthase (NOS) expression in the glomerular area and urine protein levels in streptozocin-treated rats^6^. Kodera showed that exendin-4 at 10 μg/kg daily for 8 weeks could ameliorate albuminuria, glomerular hyperfiltration, glomerular hypertrophy, and mesangial matrix expansion in the diabetic rats, as well as decreasing oxidative stress and nuclear factor-κB activation in kidney tissue^7^. A large Scandinavian Cohort Study showed that 38,731 new users of GLP-1 receptor agonists (liraglutide 92.5%, EXE 6.2%, lixisenatide 0.7%, and dulaglutide 0.6%) had a lower risk of serious renal events^8^. However, Tonneijck demonstrated that acute intravenous administration of EXE did not affect gold-standard-measured GFR and effective renal plasma flow (ERPF) in T2DM^9^.

Moreover, a retrospective analysis of an observational study showed that EXE twice daily did not change kidney function or albuminuria compared with insulin glargine. However, this observational study has some limitations in that the data capture method did not provide information on dosage or medication adherence^10^. Therefore, more robust evidence needs to be confirmed. Recently, a pooled analysis demonstrated the albuminuria-lowering effect of exenatide once weekly is partially mediated by weight loss^11^. It is necessary to evaluate the renoprotective effects of exenatide in overweight/obese patients with T2DM, to reveal the association between weight and renoprotective effects of exenatide.

In summary, we designed a randomized clinical trial experiment lasting 24 weeks to assess the renoprotective effect of EXE in overweight/obese patients with T2DM. We also evaluated the impact on glycemic control, lipid metabolism, obesity indices, systematic inflammation biomarkers and safety. Besides, we unveiled a novel role of fibroblast growth factor 21 (FGF21) in the renoprotective effect of EXE.

## Subjects, materials and methods

### Study design

The study was conducted from March to December 2017. We initially recruited the subjects at the department of endocrinology of Xinqiao Hospital, Chongqing, China. All the subjects were required to fulfill the following criteria to be eligible for our study: (1) Glycated hemoglobin (HbA1c) ≥ 6.5%; (2) Body mass index (BMI) ≥ 24 kg/m^2^ based on the criteria of the Working Group on Obesity in China^12^; (3) Age 18–65 years; (4) Systolic blood pressure (SBP) from 90 to 130 mmHg, and diastolic blood pressure (DBP) between 60 and 90 mmHg; (5) Agents such as ACE inhibitors and angiotensin receptor blockers were not allowed during the study. Exclusion criteria were as follows: cancer; cardiovascular; gastrointestinal, respiratory; kidney or liver disease diagnosis or treatment; eating disorders, psychological disorders or cognitive deficit resulting in an inability to understand or comply with instructions; lactating, pregnant, or planning pregnancy before the end of the intervention; any severe illness not otherwise specified that would interfere with the participant; smoking; alcoholism; or attending another clinical trial; lack of informed consent; and judgment of the investigator that an individual is ineligible for inclusion in the study. The clinical trial was registered at http://www.chictr.org/cn/ under study number ChiCTR-IPR-17010825 and approved by the Ethical Committee of Army Medical University. All participants signed informed consent forms before beginning the study. The study was conducted following the principles of the Declaration of Helsinki (More details of the ethics statement were shown in Supplementary Materials).

Eligible patients were randomized in equal proportions to EXE and insulin glargine group (GLAR). EXE was initiated at a dose of 5 μg b.i.d. for 4 weeks, subcutaneously injected 15 min before breakfast and dinner. Then, EXE was up-titrated to 10 μg b.i.d. for the remaining weeks^13^. Insulin glargine was chosen as the insulin comparator because it is commonly used alongside oral agents as an injectable therapy when glucose control deteriorates and is associated with a low incidence of hypoglycemia. Insulin glargine was subcutaneously injected once a day. Randomization was done centrally by computer-generated randomization lists. Sealed envelopes containing computer-generated random numbers were opened by a non–nursing staff not involved in the study on the day of visiting. The participants, investigators, and the sponsor's clinical team were blinded to treatment allocation. Patients were instructed to an average protein intake (0.8–1.2 g/kg/day) to reduce diet-induced variation in renal physiology 3 days before the visit. Besides, Patients were asked to refrain from vigorous physical activity and alcohol, caffeine, or nicotine for > 24 h before the experiments. The study protocol was deposited in protocols.io (https://doi.org/10.17504/protocols.io.bgsyjwfw) and Supplementary Material.

### Main outcomes and measures

All subjects had fasted for 8 h on the day of clinical examination. The primary outcome variable was the urine albumin concentration (UAC) measured using a Beckman Coulter UniCelDxC800 Synchron Clinical System. The microalbuminuria was defined as a 20 to 300 mg/L UAC by morning urine collection, as previously reported^14^. Secondary outcomes included: (1) Height, weight, BMI, waist-hip ratio (WHR), and visceral fat area (VFA) measurements were taken in a laboratory between 8:00 and 11:00 a.m., after a minimum of 12 h of fasting by using the In-Body (720) body composition analyzer (Biospace Co., Ltd., Seoul, Korea); (2) Fasting blood glucose (FBG) and HbA1c were measured by using a blood glucose meter (Bayer, Germany) and automatic biochemical analyzer (Hitachi 7600-020, Japan). Serum insulin was determined by an electrochemiluminescence immunoassay with an automated immunoassay analyzer (E170; Roche). C-peptide was measured by immunoradiometric assay (Centaur; Bayer Diagnostics, Mijdrecht, Netherlands). HOMA-IR values were calculated with the use of the following formula: HOMA-IR = [glucose (mmol/L) × insulin (μIU/mL)]/22.5; (3) Triglycerides (TG), total cholesterol (TCH), low-density lipoprotein cholesterol (LDL-c), high-density lipoprotein cholesterol (HDL-c) were detected by using automatic biochemical analyzer (Hitachi 7600-020, Japan); (4) C-reactive protein (CRP), interleukin-6 (IL-6), interleukin-8 (IL-8) and tumor necrosis factor-α (TNF-α) were measured by using a multiplex xMAP technology with a Luminex 100 Analyzer (Luminex Corp., Austin, TX, USA). Serum FGF21 level was measured using a Human FGF21 Quantikine ELISA Kit (R&D Systems). All of the samples were collected after ≥ 12 h of fasting. The tests are double-blind, and the quality control is carried out regularly by the inspection department.

### Dietary and physical activity measures

Twenty-four-hour dietary recalls (24-HDR) were collected and analyzed by trained staff according to procedures described in the United States Department of Agriculture (USDA) five-step multiple-pass method^15^. Participants were given the food models and food amount booklets for visualizing portion sizes and obtaining accurate estimations of foods consumed. The International Physical Activity Questionnaire Short form (IPAQ-S) was used to estimate physical activity level^16^. Participants reported the requency (days per week) and duration (time in minutes) of varying levels of physical activity over the previous 7 days. Physical activity is expressed in metabolic equivalent minutes per week (MET-min/wk). The IPAQ-S categorizes mode of activity by sitting, walking, moderate physical activity and vigorous physical activity. The total amount of physical activity performed in a week was determined by calculating the sum of moderate, vigorous, and walking MET-min/wk. All measurements were performed at the beginning and end of the study.

### Safety and tolerability assessments

Participants had low blood sugar and other adverse reactions such as nausea, vomiting, and dizziness. Patients with palpitations, sweating, and other discomforts were immediately assessed for their blood glucose levels. Hypoglycemia was defined as less than 3.9 mmol/L; Severe hypoglycemia was defined as either blood glucose levels of less than 3.1 mmol/L were observed, or hypoglycemic symptoms were relieved after oral administration of carbohydrates or intravenous glucose or glucagon.

### Statistical analyses

This study was a randomized controlled trial with two parallel groups. A sample size of 71 patients per group was required to provide 90% power to detect a between-group significant difference in UAC between the two treatment groups, assuming that the data is analyzed on a log-scale, with 0.05 alpha level and an intrasubject coefficient of variation of 50%. Moreover, we divide the alpha level by the number of tests being carried out for alpha adjustments of further analysis using 'longpower' R packages. R software (version 3.4.4) was used for statistical analysis unless otherwise stated. An intent-to-treat (ITT) criterion was performed with all participants, including those who withdrew before outcome determination. Between groups and within-group comparisons of continuous variables were conducted using linear mixed-effects models in "lmerTest" and "emmeans" packages in R. Time was treated as a categorical variable. The models included group and time as fixed covariates and the group × time interactions, with gender as covariates. Random intercepts for subjects accounted for the dependence of repeated measures^17^. To evaluate the size of the difference between and within the groups, we performed the effect size (ES) that reflects changes in SD units. Correlation-based network analysis was performed among metabolic parameters adjusted for age and gender were calculated with Pearson's correlations coefficient on residuals from regression models, including the adjustment variables for each pair of biomarkers. The Pearson correlation was used to estimate correlation coefficients. Threshold tests for correlation coefficients (r) and q-values were applied to detect significant correlations. Thresholds were set for q of ≤ 0.05. Spurious correlations were removed, while significant correlations were transformed into network form. All statistical tests were 2-tailed and, unless otherwise noted, *P* < 0.05 was considered the level of statistical significance. Variables are expressed as frequencies with percentages, median (IQRs), and means (SD).

## Results

### Participant characteristics

**Figure 1:**
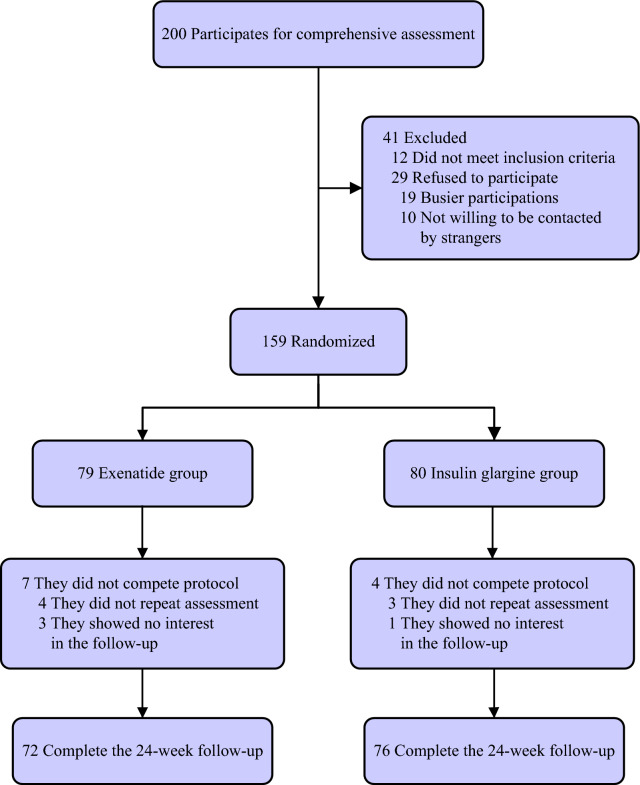
Flow diagram of the study participants.

A total of two groups were enrolled in 79 and 80, respectively. At the end of the intervention, seven patients in the EXE group and four in the GLAR group withdrew. The reason for the loss of follow-up was that the patients did not return to the outpatient department for blood sampling because of an unexpected lack of interest during the study (Fig. 1). There were no significant differences between the two groups in age, gender, duration of diabetes, prestudy antidiabetic treatment, the use of lipid-regulating drugs, and albuminuria before intervention (Table 1). At the end of the study, only two people in GLAR group started to use statins for lipid-regulating. In contrast, medication history for glucose-lowering drug and lipid-lowering drug of others did not change in both groups. Besides, macronutrient intakes by 24-HDR and physical activity measures showed no difference with-in each group and between groups (Supplementary table). After EXE treatment, 19 patients (26.39%) had mild gastrointestinal adverse reactions. One patient with moderate adverse reactions was relieved spontaneously with the treatment cycle's extension, without other adverse severe reactions.

**Table 1:** Baseline characteristics of the two groups.

Variable name	EXE (n = 79)	GLAR (n = 80)
Age (years)	49.37 ± 8.43	47.63 ± 9.65
Gender: men, no. (%)	42 (53.16)	45 (56.25)
Duration of diabetes (years)	3.0 (1.0, 5.0)	3.0 (2.0, 4.0)
**Prestudy antidiabetic treatment, no. (%)**		
Metformin and sulphonylurea combination	42 (53.16)	48 (60.00)
Sulphonylurea alone	10 (12.66)	12 (15.00)
Metformin alone	27 (34.18)	20 (25.00)
**Treatment for other risk factors, no. (%)**		
Lipid-regulating drugs	15 (18.99)	17 (21.25)
**Education, no. (%)**		
< 6 y	26 (32.91)	37 (46.25)
6–11 y	38 (48.10)	30 (37.50)
> 11 y	15 (18.99)	13 (16.25)
**Household income (¥/month), no. (%)**		
< 1000	8 (10.13)	11 (13.75)
1000–3000	28 (35.44)	28 (35.00)
3000–6000	31 (39.24)	31 (38.75)
> 6000	12 (15.19)	10 (12.50)
**Urbanity**		
Urban	30 (37.97)	28 (35.00)
Rural	49 (62.03)	52 (65.00)
**UAC**		
Median (IQRs), mg/L	80.0 (50.0, 150.0)	80.0 (40.0, 108.8)
Geometric mean (CV%), mg/L	80.60 (84.86%)	69.59 (86.94%)
**Albuminuria, no. (%)**		
Normal (≤ 20 mg/L)	5	7
High (> 20– < 300 mg/L)	73	72
Very high (≥ 300 mg/L)	1	1

Data are presented as frequencies with percentages, median (IQRs), and mean ± SD.

UAC, urine albumin concentration.

### Primary end point

**Figure 2:**
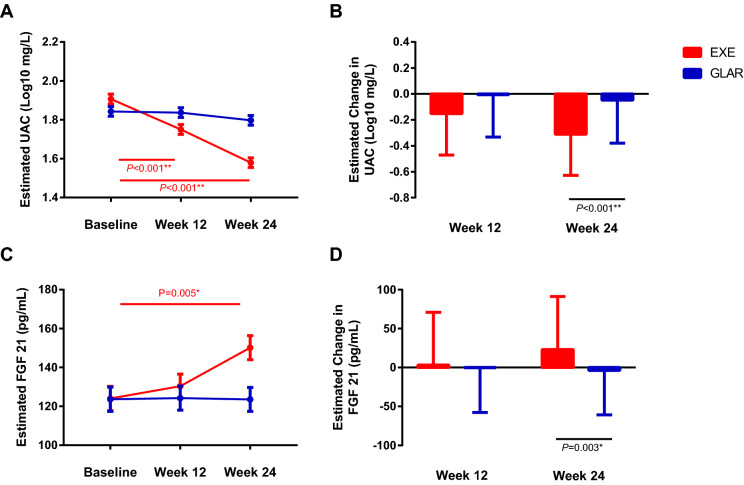
Comparison of changes in UAC and FGF 21 within and between the groups. The linear mixed-effects model was used to compare within-group and intergroup changes of UAC (**A**,**B**) and FGF 21 (**C**,**D**) with gender as covariant. Data are presented as means ± SD., *P < 0.01, **P < 0.001.

We conducted analyses to identify differences within and between groups of UAC during the study periods. EXE intervention could significantly reduce the UAC at week 12 and week 24 endpoints (*P* < 0.001, Fig. 2A), while Insulin glargine did not significantly influence the UAC. Linear mixed-effects models analysis revealed that subjects in the EXE group had significantly improved UAC than those in the GLAR group at week 24 (*P* < 0.001, Fig. 2B).

### Other efficacy variables

The anthropometric parameters (weight, BMI, WHR, VFA) and glucose parameters and biomarkers of lipid profile and inflammation biomarkers (CRP, TNF-α and Il-6) showed no significant differences between the two groups at baseline. The anthropometric parameters (weight, BMI, WHR, VFA, *P* < 0.001 for all parameters at week 12 and week 24), glucose parameters (FBG, HbA1c, insulin and HOMA-IR, *P* < 0.001 for all parameters at week 12 and week 24), lipid parameters (TG: *P* = 0.020 at week 24; HDL-c: *P* = 0.049 at week 24), and inflammation biomarkers (CRP: *P* < 0.001 at week 24; TNF-α: *P* < 0.001 at week 12 and week 24) were significantly improved at both week 12 or week 24 endpoints in EXE group (Table 2). Meanwhile, the comparison between groups showed that changes of anthropometric parameters (Weight: *P* = 0.002 at week 24; BMI: *P* < 0.001 at week 24; WHR: *P* = 0.008 at week 24; VFA: P < 0.001 at week 12 and week 24), glucose parameters (FBG: *P* < 0.001 at week 12 and *P* = 0.001 at week 24; HbA1c: *P* < 0.001 at week 12; C-peptide: *P* < 0.001 at week 12 and week 24; HOMA-IR: *P* = 0.004 at week 12 and *P* = 0.040 at week 24), lipid parameters (TG: *P* = 0.002 at week 12 and *P* = 0.012 at week 24; HDL-c: *P* = 0.001 at week 12 and *P* = 0.028 at week 24), and biomarkers of inflammation profile (CRP: *P* = 0.002 at week 12 and *P* < 0.001 at week 24; IL-6: *P* = 0.020 at week 24; TNF-α: *P* < 0.001 at week 12 and week 24) of patients in the EXE group were significantly different from those in another group at week 12 or week 24 (Table 2).

**Table 2:** Baseline value and estimate change in the anthropometric composition parameters, biochemical variables and inflammation biomarkers in two groups at 12 and 24 weeks.

	ITT analysis of EXE within group			ITT analysis of GLAR within group			Between group		
	Mean (SD)	*P-*value	ES	Mean (SD)	*P*-value	ES	*P*-value	95%CI	ES
**Anthropometric parameters**									
Weight (kg)									
Baseline	75.241 (8.167)			73.575 (6.106)			0.123	− 0.312 to 2.585	1.136
∆ week 12	− 2.969 (8.148)	< 0.001	2.187	− 0.414 (6.043)	0.141	0.302	0.309	− 2.197 to 0.701	− 0.748
∆ week 24	− 5.388 (8.334)	< 0.001	3.720	− 0.658 (5.915)	0.221	0.269	0.002	− 3.773 to − 0.857	− 2.315
BMI, kg/ m^2^									
Baseline	27.607 (2.027)			27.064 (1.937)			0.084	− 0.132 to 2.056	0.962
∆ week 12	− 1.160 (2.041)	< 0.001	2.204	− 0.120 (1.897)	0.130	0.308	0.094	− 2.029 to 0.163	− 0.933
∆ week 24	− 2.005 (2.078)	< 0.001	3.734	− 0.060 (1.882)	0.200	0.277	< 0.001	− 3.603 to − 1.385	− 2.494
WHR									
Baseline	0.901 (0.041)			0.898 (0.032)			0.578	− 0.421 to 0.752	0.166
∆ week 12	− 0.015 (0.039)	< 0.001	0.744	− 0.007 (0.033)	0.046	0.379	0.508	− 0.789 to 0.392	− 0.199
∆ week 24	− 0.031 (0.039)	< 0.001	1.628	− 0.010 (0.033)	0.000	0.653	0.008	− 1.406 to − 0.212	− 0.809
VFA (m^2^)									
Baseline	104.667 (25.236)			104.075 (17.198)			0.856	− 0.656 to 0.789	0.067
∆ week 12	− 14.492 (24.636)	< 0.001	1.721	− 0.976 (17.211)	0.849	0.087	< 0.001	− 2.301 to − 0.834	− 1.568
∆ week 24	− 24.912 (24.482)	< 0.001	2.869	− 3.117 (17.511)	0.102	0.331	< 0.001	− 3.219 to − 1.724	− 2.471
**Parameter of glucose metabolism**									
FBG (mmol/L)									
Baseline	9.916 (2.157)			9.578 (1.633)			0.164	− 0.116 to 0.678	0.281
∆ week 12	− 2.338 (2.154)	< 0.001	1.845	− 1.007 (1.645)	< 0.001	0.826	< 0.001	− 1.143 to − 0.333	− 0.738
∆ week 24	− 3.063 (2.136)	< 0.001	2.385	− 1.780 (1.658)	< 0.001	1.419	0.001	− 1.094 to − 0.275	− 0.685
HBA1C (%)									
Baseline	9.726 (1.974)			9.644 (1.545)			0.874	− 0.301 to 0.3541	0.027
∆ week 12	− 1.760 (2.016)	0.001	0.580	0.184 (1.550)	0.930	− 0.058	< 0.001	− 0.947 to − 0.276	− 0.612
∆ week 24	− 2.574 (2.020)	< 0.001	0.823	− 1.562 (1.513)	0.004	0.515	0.103	− 0.620 to 0.0573	− 0.282
C-peptide (nmol/L)									
Baseline	1.352 (0.452)			1.309(0.386)			0.538	− 0.239 to 0.458	0.109
∆ week 12	0.652 (0.427)	< 0.001	− 1.556	0.151 (0.381)	0.065	− 0.357	< 0.001	0.944 to 1.672	1.308
∆ week 24	1.039 (0.427)	< 0.001	− 2.431	0.338 (0.388)	< 0.001	− 0.780	< 0.001	1.383 to 2.138	1.761
Insulin (mU/L)									
Baseline	9.949 (3.080)			9.491 (2.920)			0.198	− 0.115 to 0.552	0.218
∆ week 12	− 1.723 (3.112)	< 0.001	0.768	− 0.473 (2.940)	0.364	0.216	0.053	− 0.673 to 0.005	− 0.334
∆ week 24	− 2.812 (3.145)	< 0.001	1.282	− 1.988 (2.990)	< 0.001	0.935	0.461	− 0.473 to 0.215	− 0.129
HOMA-IR									
Baseline	4.320 (1.620)			4.056 (1.547)			0.141	− 0.083 to 0.5808	0.249
∆ week 12	− 1.488 (1.618)	< 0.001	1.318	− 0.621 (1.561)	0.001	0.571	0.004	− 0.836 to − 0.159	− 0.497
∆ week 24	− 2.190 (1.624)	< 0.001	1.947	− 1.466 (1.585)	< 0.001	1.340	0.040	− 0.702 to − 0.015	− 0.359
**Parameter of lipid metabolism**									
TCH (mmol/L)									
Baseline	4.863 (1.012)			4.952 (0.850)			0.558	− 0.410 to 0.222	− 0.094
∆ week 12	− 0.198 (0.980)	0.604	0.155	− 0.125 (0.842)	0.655	0.139	0.502	− 0.431 to 0.212	− 0.110
∆ week 24	− 0.230 (0.970)	0.564	0.166	0.022 (0.857)	0.999	− 0.007	0.109	− 0.594 to − 0.060	− 0.267
TG (mmol/L)									
Baseline	2.037 (1.289)			1.974 (1.131)			0.758	− 0.264 to 0.362	0.049
∆ week 12	0.076 (1.294)	0.783	− 0.108	− 0.463 (1.134)	0.066	0.356	0.002	0.193 to 0.832	0.513
∆ week 24	0.471 (1.300)	0.020	− 0.438	0.048 (1.144)	0.893	− 0.073	0.012	0.090 to 0.739	0.414
LDL-c (mmol/L)									
Baseline	2.745 (0.698)			2.596 (0.706)			0.145	− 0.080 to 0.544	0.232
∆ week 12	− 0.168 (0.665)	0.417	0.204	0.167 (0.704)	0.203	− 0.271	0.132	− 0.561 to 0.074	− 0.243
∆ week 24	− 0.164 (0.685)	0.358	0.222	0.104 (0.665)	0.337	− 0.226	0.187	− 0.539 to 0.106	− 0.217
HDL-c (mmol/L)									
Baseline	1.389 (0.452)			1.431 (0.329)			0.552	− 0.452 to 0.242	− 0.105
∆ week 12	− 0.097 (0.461)	0.287	0.244	0.099 (0.329)	0.233	− 0.259	0.001	− 0.964 to − 0.254	− 0.609
∆ week 24	0.168 (0.458)	0.049	− 0.386	− 0.058 (0.325)	0.727	0.122	0.028	0.044 to 0.762	0.403
**Inflammation biomarkers**									
CRP (mg/L)									
Baseline	4.927 (2.897)			5.291 (2.229)			0.331	− 0.643 to 0.218	− 0.213
∆ week 12	− 0.321 (2.818)	0.394	0.212	0.435 (2.192)	0.205	− 0.271	0.002	− 1.132 to − 0.257	− 0.695
∆ week 24	− 2.406 (2.816)	< 0.001	1.452	0.401 (2.221)	0.291	− 0.242	< 0.001	− 2.364 to − 1.448	− 1.906
IL-6 (pg/mL)									
Baseline	4.311 (2.494)			4.489 (1.817)			0.591	− 0.447 to 0.255	− 0.096
∆ week 12	0.254 (2.523)	0.515	− 0.178	0.211 (1.828)	0.666	− 0.137	0.763	− 0.411 to 0.302	− 0.055
∆ week 24	− 0.311 (2.545)	0.691	0.134	0.296 (1.841)	0.430	− 0.199	0.020	− 0.792 to − 0.066	− 0.429
IL-8 (pg/mL)									
Baseline	7.265 (1.473)			7.059 (1.187)			0.413	− 0.218 to 0.530	0.156
∆ week 12	0.183 (1.489)	0.727	− 0.123	− 0.053 (1.191)	0.962	0.042	0.097	− 0.059 to 0.701	0.321
∆ week 24	0.292 (1.500)	0.446	− 0.198	0.278 (1.145)	0.2266	− 0.250	0.594	− 0.281 to 0.489	0.104
TNF-α (pg/mL)									
Baseline	4.856 (1.719)			4.748 (1.572)			0.711	− 0.324 to 0.475	0.075
∆ week 12	1.616 (1.715)	< 0.001	− 1.196	0.059 (1.565)	0.925	− 0.060	< 0.001	0.800 to 1.624	1.212
∆ week 24	2.882 (1.599)	< 0.001	− 2.017	0.164 (1.586)	0.721	− 0.124	< 0.001	1.539 to 2.398	1.969

Data are presented as mean (SD). Between-group and Within-group comparisons were analyzed by Linear mixed-effects model analysis.

BMI, body mass index; WHR, waist-hip ratio; VFA, visceral fat area; FBG, fasting blood glucose; HbA1C, glycosylated hemoglobin; TCH, total cholesterol; TG, triglycerides; LDL-c, low-density lipoprotein cholesterol, HDL-c, high-density lipoprotein cholesterol; CRP, C-reactive protein; IL-6, interleukin-6; IL-8, interleukin-8; TNF-α, tumor necrosis factor-α.

### Correlations between FGF 21 and other efficacy variables

To explore the mechanism of the renoprotective effect of EXE, we measured the level of serum FGF21. EXE significantly increased FGF 21 at week 24 (*P* = 0.005, Fig. 2C). Between-group comparison uncovered that EXE group subjects had significantly improved FGF 21 level than those in the GLAR group at week 24 using linear mixed-effects models analysis (*P* = 0.003, Fig. 2D).

**Figure 3:**
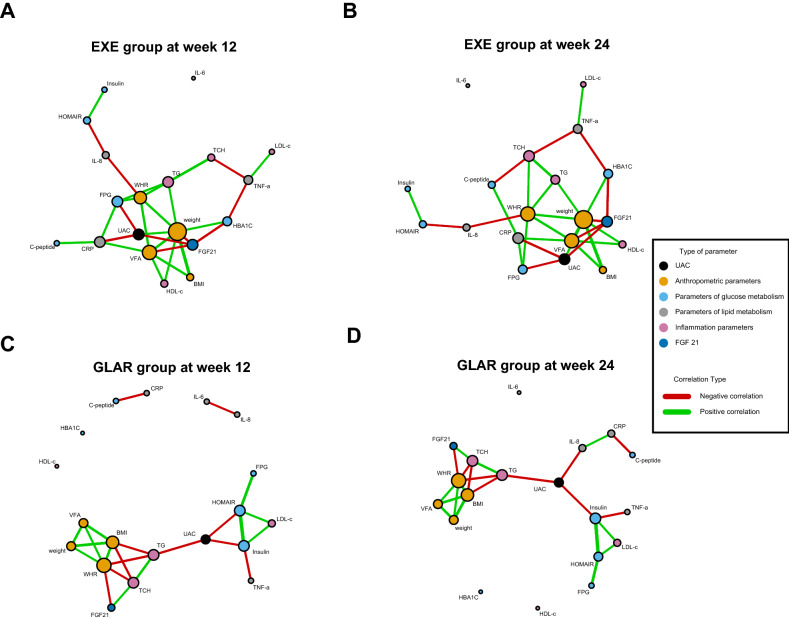
Correlation-based network analysis. Network visualization of correlations in the EXE group (**A**,**B**) and the GLAR group (**C**,**D**) at week 12 and 24. Metabolic parameters are displayed as nodes and color-coded according to the type of parameters. UAC, anthropometric parameters, glucose and lipid metabolism parameters, inflammation parameters, and FGF 21 are displayed as black, yellow, blue, grey, pink, and dark blue nodes. The thickness of edges linking different variables represents significant positive (green) or negative (red) correlation coefficients. UAC, urine albumin concentration; BMI, body mass index; WHR, waist-hip ratio; VFA, visceral fat area; FBG, fasting blood glucose; HbA1c, glycosylated hemoglobin; TCH, total cholesterol; TG, triglycerides; LDL-c, low-density lipoprotein cholesterol, HDL-c, high-density lipoprotein cholesterol; CRP, C-reactive protein; IL-6, interleukin-6; IL-8, interleukin-8; TNF-α, tumor necrosis factor-α; FGF21, fibroblast growth factor 21.

We then explored correlation-based network analysis among UAC, anthropometric parameters, glucose and lipid metabolism parameters, inflammation parameters, and FGF 21. The overall results demonstrated more associations between FGF 21 and other metabolic parameters in the EXE group when compared with the GLAR group (Fig. 3). Detailed correlation analysis showed that the estimated change of FGF 21 was negatively correlated with UAC (Week 12: *r* = − 0.297, *P* = 0.010; Week 24: *r* = − 0.294, *P* = 0.012), Weight (Week 12: *r* = − 0.336, *P* = 0.003; Week 24: *r* = − 0.337, *P* = 0.004), VFA (Week 12: *r* = − 0.273, *P* = 0.018; Week 24: *r* = − 0.281, *P* = 0.016) and HbA1c (Week 12: *r* = − 0.340, *P* = 0.003; Week 24: *r* = − 0.365, *P* = 0.002) in EXE group at both week 12 and week 24 (Supplementary Figure A,B). However, a few correlations between FGF 21 and WHR or TCH was found in the GLAR group (Supplementary Figure C,D). Besides, there was an association between the estimated change of weight and UAC (Week 12: r = 0.264, *P* = 0.022; Week 24: r = 0.253, *P* = 0.031, Supplementary Figure A,B), indicating that weight loss is mediated the UAC-lowering effect of EXE. Together, these results indicated that FGF 21 might play an important role in improving EXE on urine albumin and other metabolic parameters.

## Discussion

We completed a 24-week, prospective, randomized controlled trial of the EXE intervention in overweight/obese patients with T2DM. The main finding of our study was that 24-week EXE intervention significantly reduced UAC, which is significantly correlated with FGF 21. The results also showed that the patients in the EXE group presented more significant improvements in weight and glucose parameters (FBG, HbA1c). These data indicated that using EXE among overweight/obese patients with T2DM is more helpful than traditional outpatient services for better UAC and glycemic control.

Abdominal obesity can lead to glucose metabolism disorders and atherosclerosis, which significantly impacts the metabolic level and leads to insulin resistance^18^. Our results showed that a decrease in UAC is positively correlated with the change of WHR, indicating that abdominal obesity is more closely related to DN than systemic obesity. Furthermore, as reported previously, the correlation between abdominal obesity and DN is independent of known risk factors such as BMI and age, diabetes course, and blood pressure^1,19,20^. Weight loss could also reduce urinary albumin excretion and prevent glomerular filtration in obese patients with DN^21^. Besides, a pooled analysis contains six randomized trials showed that adjustment for concomitant changes in body weight reduced the urinary albumin-to-creatinine ratio (UACR)-lowering effect of exenatide compared to the comparator group, indicating that body weight is also an essential factor for microalbuminuria in T2DM^11^. Therefore, losing weight, especially improving abdominal obesity and lowering blood glucose, is significant in delaying DN development. We also found that the serum levels of TG and HDL-c were improved accordantly with the previous result^22^. However, Tch and LDL-c levels were not changed, which may be due to either the small sample size or the duration of intervention, or both. This study used WHR and VFA to assess abdominal obesity and found that EXE dramatically improved abdominal obesity, which positively correlates with UAC.

Microalbuminuria is the earliest biochemical parameter of DN and the important risk factor affecting DN progression and cardiovascular diseases. In line with previous studies, our results showed that EXE dramatically improved UAC, a well-known biomarker of microalbuminuria^23^. EXE is a synthetic version of exendin-4 and displays biological properties similar to human GLP-1, produced by intestinal L endocrine cells when ingesting food^4^. A previous study showed that Exendin-4 ameliorated albuminuria and glomerular hyperfiltration in diabetic rats by decreasing oxidative stress and nuclear factor-κB activation in kidney tissue. Also, it reduced pro-inflammatory cytokines from macrophages on glomerular endothelial cells^7^. After 16 weeks of treatment in T2DM patients, EXE attenuated urinary excretion of TGF-1 and type IV collagen, which has been proven to correlate with microalbuminuria severity^23^. The mRNA expression of TNF-α, IL-1β, JNK-1, TLR-2, TLR-4, and SOCS-3 in mononuclear cells and monocyte plasma concentrations chemoattractant protein-1, matrix metalloproteinase-9, serum amyloid A, and IL-6 of 12 patients was significantly decreased after 12 weeks of EXE treatment^24^. Dandona reported that EXE increased the Interleukin-1 receptor antagonist (IL-1RA) via the Nrf2-Keap1-ARE system to suppress inflammatory and oxidative stress^25^. Moreover, activation of Nrf2-Keap1 could suppress oxidative stress to protect renal function^26^. These results indicated that EXE could improve urinary microalbuminuria by reducing inflammation and oxidative stress, suggesting a pioneering role of EXE in renal protection and against the progression of DN. However, few clinical studies evaluated the impact of EXE on inflammation. Here, we identified that EXE could reduce inflammation parameters while improving microalbumin in overweight/obese patients with T2DM.

Numerous studies have uncovered that FGF21 causes considerable pharmacological benefits on metabolic diseases, such as hyperglycemia, insulin resistance, dyslipidemia, and other obesity-related disorders^27^. Here, our results showed that EXE could significantly increase the serum level of FGF 21, which is negatively correlated with the change of UAC. Liu showed that FGF 21 is upregulated by EXE in both hepatocytes and humans, and the increase of FGF21 was blocked in *Fgf21* knockout mice^28^. Nevertheless, no study has assessed the correlation between FGF 21 and the renoprotective effect of EXE in humans. And our results firstly showed that FGF 21 may have strong links between weight loss and UAC-lowering effect of EXE.

A previous meta-analysis study concluded that both UAC and UACR yielded high sensitivity and specificity to detect microalbuminuria in patients with diabetes mellitus after collecting and carefully analyzing 14 published studies^29^. Here, we used UAC as the biomarker for microalbuminuria due to its convenient use and low cost for patients. Moreover, due to the limitation of the study period, whether the improvement in urinary microalbumin could be maintained needs further evaluation. Likewise, whether the VFA and weight of patients would rise for a short time after the withdrawal of EXE or the necessity of changes in hypoglycemic regimentation needs to be further evaluated.

In conclusion, our results showed that EXE could help patients improve UAC and glycemic levels and inflammatory biomarkers after a follow-up period of 24 weeks intervention, indicating that EXE is the effective and safe for albuminuria control in diabetic patients. We first showed that the increase of FGF 21 correlates with the decrease of UAC after EXE intervention, providing that FGF 21 may be involved in the renoprotective effect of EXE. Our results could help the clinician to pay more attention to the renoprotective effects of EXE when treating diabetes, especially those with obesity. These findings may provide new options for patients with T2DM seeking better albuminuria and glycemic control.

## Supplementary Information

Supplementary Information 1.

Supplementary Information 2.

Supplementary Information 3.

Supplementary Information 4.
