# Supplementary material for: Effects of exenatide on urinary albumin in overweight/obese patients with T2DM: a randomized clinical trial
Source: Sci Rep. 2021 Oct 8;11:20062. doi: 10.1038/s41598-021-99527-y (PMC8501012; doi:10.1038/s41598-021-99527-y)
Supplement: Supplementary file 1 — Supplementary Information 1. [file 41598_2021_99527_MOESM1_ESM.pdf]

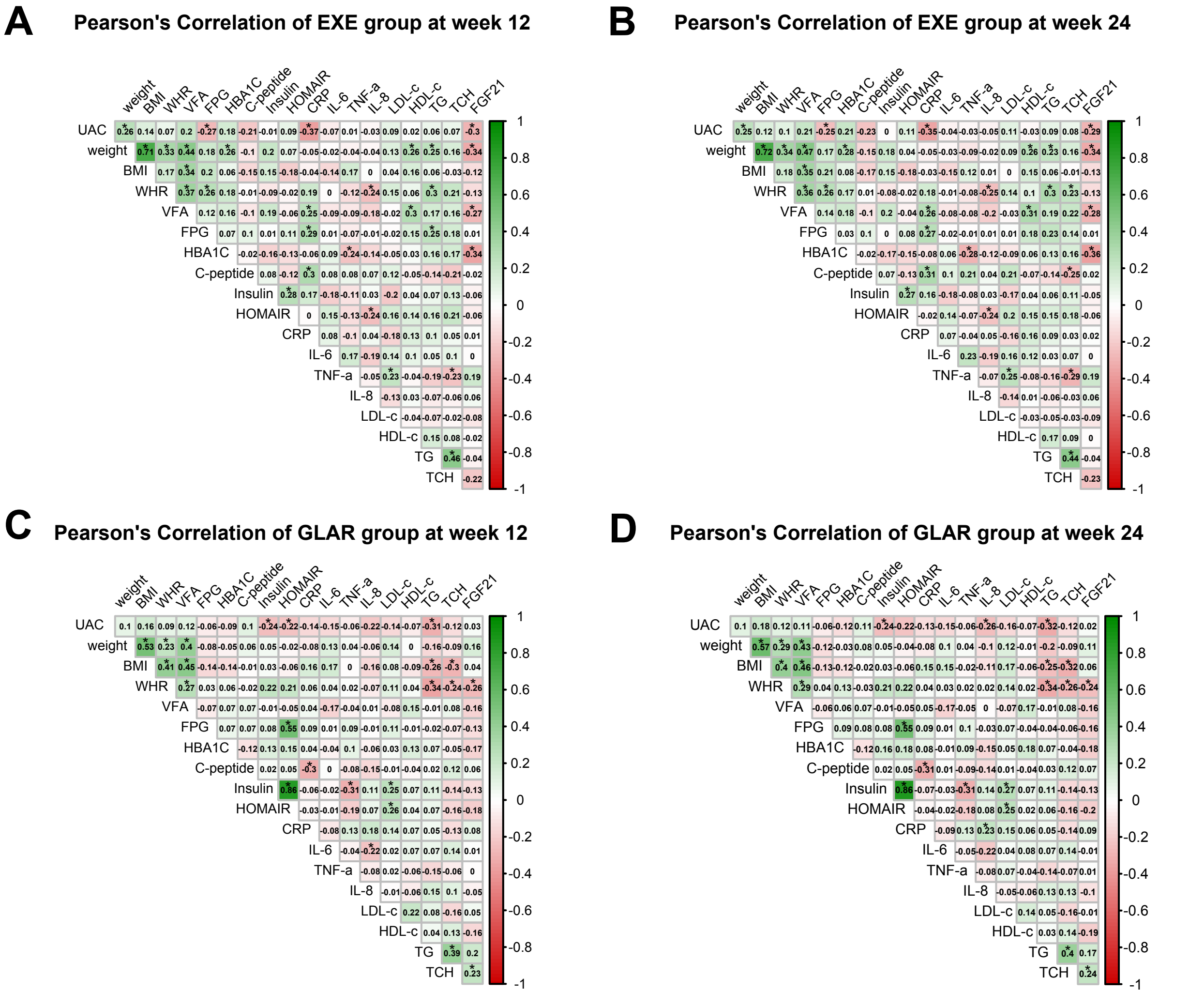

Supplementary Figure. Heatmap displaying Pearson's correlations among metabolic parameters in the EXE group (A-B) and the GLAR group (C-D) at week 12 and 24. Pearson's correlation coefficients (*r*) and the corresponding *P*-values are presented. **P* <0.05. Cell colors range from positive correlation (green) to negative correlation (red).

Abbreviations: UAC, urine albumin concentration; BMI, body mass index; WHR, waist-hip ratio; VFA, visceral fat area; FBG, fasting blood glucose; HbA1c, glycosylated hemoglobin; TCH, total cholesterol; TG, triglycerides; LDL-c, low-density lipoprotein cholesterol, HDL-c, high-density lipoprotein cholesterol; CRP, C-reactive protein; IL-6, interleukin-6; IL-8, interleukin-8; TNF-α, tumor necrosis factor-α; FGF21, fibroblast growth factor 21.
