# Supplementary material for: Effects of exenatide on urinary albumin in overweight/obese patients with T2DM: a randomized clinical trial
Source: Sci Rep. 2021 Oct 8;11:20062. doi: 10.1038/s41598-021-99527-y (PMC8501012; doi:10.1038/s41598-021-99527-y)
Supplement: Supplementary file 2 — Supplementary Information 2. [file 41598_2021_99527_MOESM2_ESM.docx]

**Supplementary Table. Macronutrient intakes by 24-hour dietary recall and physical activity measures.**

| **Variable name** | **EXE (n=79)** | | | **GLAR (n=80)** | | | ***P* value**^b^ |
| --- | --- | --- | --- | --- | --- | --- | --- |
|  | **Baseline** | **24 week** | ***P* value**^a^ | **Baseline** | **24 week** | ***P* value**^a^ |  |
| Energy intake (kcal) | 2672.535±573.026 | 2855.479±485.776 | 0.354 | 2867.545±516.601 | 2987.272±400.681 | 0.484 | 0.803 |
| % kcal from carbohydrate | 61.910±7.344 | 61.133±7.214 | 0.773 | 62.400±9.378 | 59.881±7.477 | 0.423 | 0.618 |
| % kcal from protein | 14.142±1.705 | 13.668±1.995 | 0.490 | 14.177±2.059 | 13.916±2.736 | 0.770 | 0.805 |
| % kcal from fat | 27.892±6.289 | 28.760±6.322 | 0.709 | 27.916±8.158 | 31.290±5.938 | 0.206 | 0.453 |
| Physical activity (total MET-min/wk) | 3559.393±2988.366 | 3872.372±3134.636 | 0.782 | 4192.705±4350.028 | 3619.691±4504.524 | 0.726 | 0.438 |

Data are presented as mean±SD.

Abbreviations: MET, metabolic equivalent.

^a^ Within-group differences from baseline to 24 week.

^b^ Differences between groups from baseline to 24 week.
