# Supplementary material for: Effects of exenatide on urinary albumin in overweight/obese patients with T2DM: a randomized clinical trial
Source: Sci Rep. 2021 Oct 8;11:20062. doi: 10.1038/s41598-021-99527-y (PMC8501012; doi:10.1038/s41598-021-99527-y)
Supplement: Supplementary file 3 — Supplementary Information 3. [file 41598_2021_99527_MOESM3_ESM.doc]

**Study Protocol**

| Name | | Effects of exenatide on urinary albumin in overweight/obese patients with type 2 diabetes mellitus: a randomized clinical trial | | | | |
| --- | --- | --- | --- | --- | --- | --- |
| Applying Department | | Department of Endocrinology, Xinqiao Hospital | | | | |
| Version | | 2.0 | | Date | | 2016.05.09 |
| **Content** | | | | | | |
| **Background**  Among the Chinese adults with diabetes, the proportion of overweight is 41.0%, the proportion of obesity is 24.3%, and the proportion of abdominal obesity is as high as 45.4% in 2008, which shows an increasing trend in recent years. Clinical studies have demonstrated that Asian people have higher levels of visceral fat than European and American populations. Obesity, especially abdominal obesity is closely associated with Diabetic Nephropathy (DN). exenatide improves glycemic control and reduces body weight in patients with T2DM. In animal studies, exenatide can ameliorate albuminuria and improve kidney function. However, only a few studies evaluate the microalbuminuria in human. This study aims to investigate the effect of exenatide on urinary albumin, obese indices and glucose control in overweight/obese patients with type 2 diabetes.  **Study Plan**  (1) 2016.7~2017.2 Search related papers online and make a clinical protocol.  (2) 2017.3~2017.9 Recruit volunteers, follow-up and collect samples.  (3) 2017.10~2017.12 Data analysis and writing papers.  **Inclusion Criteria**  (1) Eligible subjects had been newly diagnosed within the past 3 mo according to the diagnostic criteria of T2DM, which glycated hemoglobin (HbA1c) of 6.5–7.5%; (2) BMI≥24; (3) Age 18–65 y; (4) Systolic blood pressure (SBP) from 90 to 120 mm Hg, and diastolic blood pressure (DBP) between 60 and 90 mm Hg; (5) Agents such as ACE inhibitors and angiotensin receptor blockers were not allowed during the study.  **Exclusion Criteria**  Cancer; cardiovascular; gastrointestinal, respiratory; kidney or liver disease diagnosis or treatment; eating disorders, psychological disorders or cognitive deficit resulting in an inability to understand or comply with instructions; lactating, pregnant, or planning pregnancy before the end of the intervention; any serious illness not otherwise specified that would interfere with participant; smoking; alcoholism; or attending another clinical trial; lack of informed consent; and judgment of the investigator that an individual is ineligible for inclusion in the study.  **Trial design**  Randomized Controlled Trial.  **Intervention group**  For Exenatide group, Exenatide was initiated at a dose of 5 μg b.i.d., subcutaneously injected 15 min before breakfast and dinner for 4 weeks. Then, exenatide was up-titrated to 10 μg b.i.d. for the remaining weeks of the study.  **Control group**  Subjects of control group was subcutaneously injected by insulin glargine once a day, 15 min before breakfast and dinner.  **Endpoints**  **Primary outcome**  urine albumin concentration (UAC)  **Secondary outcomes**   1. Body composition parameters (Weight, body mass index [BMI], waist-hip ratio [WHR] and visceral fat area [VFA]); 2. Fasting blood glucose (FBG), glycosylated hemoglobin (HbA1C), C-peptide, serum insulin and HOMA-IR values; 3. Inflammation parameters (C-reactive protein [CRP), interleukin-6 [IL-6] and tumor necrosis factor-α [TNF-α]).   **Sample size**  This study was two groups of randomized controlled trials. A sample size of 71 patients per group was required to provide 90% power to detect a between group significant difference in UAC between the two treatment groups, assuming that the data is analyzed on a log-scale, with 90% power using a two-sided independent t-test at a 0.05 alpha level.  **Feasible analysis**   1. All the drugs in this study have been widely used in clinical practice. 2. The routine outpatient service for diabetes patients in the undergraduate department can meet   the subjects' screening conditions.   1. The indexes of human body composition can be measured in the nutrition department of our hospital. The other biochemical parameters can be completed with the help of our laboratory.   (4) The outpatient visiting time of the main researcher can accomplish the investigation and follow-up of the visiting period.  **Acknowledgments**  Guarantor of the article: Jing Xu  **Funding**  This research was supported by Project 2014YLC20 of the Xinqiao Hospital, Project ctstc2015shmszx120014 and ctstc2015jcsf10003 of the Chongqing Science and Technology Bureau.  **Disclosure**  The authors have no conflicts of interest to disclose. | | | | | | |
| Signature | Jing Xu | | Date | | 05.09.2016 | |
