# Supplementary material for: Effects of exenatide on urinary albumin in overweight/obese patients with T2DM: a randomized clinical trial
Source: Sci Rep. 2021 Oct 8;11:20062. doi: 10.1038/s41598-021-99527-y (PMC8501012; doi:10.1038/s41598-021-99527-y)
Supplement: Supplementary file 4 — Supplementary Information 4. [file 41598_2021_99527_MOESM4_ESM.pdf]

Ethic Statement

English Version

中国人民解放军第三军医大学<br>第二附属医院医学伦理委员会	Medical Ethics Committee of Second Affiliated<br>Hospital of Third Military Medical University, PLA

No. AFSC08/1.0

Research Ethic Approval of Xinqiao Hospital (Formerly name: Second Affiliated Hospital of Third Military Medical University) ethics committee

Approval Document No.	16-03502		
Project Title	Effects of exenatide on urinary albumin in overweight/obese patients with type 2 diabetes mellitus: a randomized clinical trial		
Project Source	Xinqiao Hospital, Army Medical University		
Project Source	Department of Endocrinology	Principal Investigator	Jing Xu
Review Category	Recheck	Review Mode	Meeting review
Review Date	2016.12.27		
Location	Ethics Committee office, 3rd floor, Department of Pharmacy, Second Affiliated Hospital, Third Military Medical University		
Ethic Committee member	Jianchen Xu		
Review documents	Application for Ethic Approval.<br>Results of Ethic Approval.<br>Study protocol (version 2.0, 05/09/2016).<br>Informed Consent (version 2.0, 05/09/2016).		
Decision	Agree. This document is valid for one year, expiring on December 26, 2017.		
Notes	1. Clinical studies must be followed the principles of GCP, the declaration of Helsinki and the ethic approval by the ethics committee to protect the health and rights of the volunteers.<br>2. Please complete the registration or approval procedures of the clinical study as required by relevant functional departments or agencies before the study.<br>3. If the main researcher is changed or any modification is made to the program, consent form, recruitment materials and other important materials, please submit the application for amendment for review during the study.<br>4. In case of serious adverse events, please timely submit the report of serious adverse events<br>5. If the ethics committee has specified the frequency of follow-up review, please submit the research progress report one month before the deadline; As the group leader of the multi-center research project, the center shall also submit the summary report of the research progress of each center;		

	Please submit a written report to the ethics committee in a timely manner in the event of any circumstances that may significantly affect the conduct of the trial or increase the risk to the subject.<br>6. Please submit a report when study does not followed the protocol which may harm the health and rights of subjects, or have violated of GCP principles. Such as: Subjects who do not meet the Inclusion/Exclusion criteria, compliance with the discontinuation protocol without withdrawing from the study, given wrong therapy or drug dose, given prohibited drug combination solution.<br>7. If the clinical study is suspended or terminated in advance, please submit the suspension/termination report in time.<br>8. Please submit the study completion report after completing the clinical study.<br>9. This clinical trial shall be carried out within 1 year from the date of approval. If it is not carried out within the time limit, this approval document shall be abolished by itself.		
Proclaim	The ethics committee shall review the materials in Chinese version submitted for review in strict accordance with the Chinese GCP, CH-GCP and relevant laws and regulations (the CD-ROM and materials in foreign language submitted for review shall not be reviewed but recorded), and its review process shall not be influenced by any organization or individual.		
Name of Ethic Committee	Medical Ethics Committee of Second Affiliate Hospital of Third Military Medical University (stamp)		
Contact information of Ethic Committee	Fan Deng, 023-68755422		
Address of Ethic Committee	Ethics Committee office, 3rd floor, Department of Pharmacy, Second Affiliated Hospital, Third Military Medical University		
Signature	Jiancheng Xu	Date	2017.01.04

Chinese Version

文件编号：AF/SC-08/1.0

审查批件

伦理审查编号	2016-研第 035-02		
项目名称	GLP-1 受体激动剂对超重肥胖 T2DM 患者体质改善临床研究		
项目来源	自选课题		
临床研究机构	内分泌科	主要研究者	徐静
审查类别	初审后的审查	审查方式	会议审查
审查日期	2016 年 12 月 27 日		
审查地点	第三军医大学第二附属医院药学部三楼会议室		
审查委员	见附件"会议签到表"		
送审文件	复审申请<br>伦理审查意见副本<br>研究方案变更说明表<br>知情同意书变更说明表<br>修正后的临床研究方案（版本 2.0/2016 年 5 月 9 日）<br>修正后的知情同意书（版本 2.0/2016 年 5 月 9 日）		
审查决定	同意。跟踪审查频率：12 个月，截止日期为 2017 年 12 月 26 日		
注意事项	1、请遵循 GCP 原则、赫尔辛基宣言和伦理委员会批准的方案开展临床研究，保护受试者的健康与权益。<br>2、研究开始前，请按有关职能部门或机关的要求完成临床研究的注册或立项手续。<br>3、研究过程中若变更主要研究者或对方案、知情同意书、招募材料等重要材料的任何修改，请提交修正案审查申请。<br>4、本中心若发生严重不良事件，请及时提交严重不良事件报告。<br>5、伦理委员会有规定跟踪审查频率的项目，请按照跟踪审查频率，在截止日期前 1 个月提交研究进展报告；我中心作为多中心研究项目组长单位的，还应提交各中心研究进展的汇总报告；当出现任何可能显著影响试验进行或增加受试者风险的情况时，请及时向伦理委员会提交书面报告。<br>6、研究过程中若出现纳入了不符合纳入标准或符合排除标准的受试		

	者、符合中止试验规定而未让受试者退出研究、给予错误治疗或剂量、给予方案禁止的合并用药等没有遵从方案开展研究的情况或可能对受试者的权益/健康以及研究的科学性造成不良影响等违背 GCP 原则的情况，请提交违背方案报告。<br>7、暂停或提前终止临床研究，请及时提交暂停/终止研究报告。<br>8、完成临床研究，请提交研究完成报告。<br>9、本临床试验应在批准之日起 1 年内实施，逾期未实施的，本批件自行废止。
声明	本伦理委员会严格按照中国 GCP、ICH-GCP 和有关法规对送审的中文纸质材料进行评审（对送审的光盘及外文纸质材料不作评审仅备案），其审查工作过程不受伦理委员会以外的任何组织及个人的影响。
伦理委员会名称	中国人民解放军第三军医大学第二附属医院医学伦理委员会（盖章）
伦理委员会联系方式	伦理委员会办公室联系人：邓[illegible] 023-68755422
伦理委员会地址	第三军医大学第二附属医院药学部三楼伦理委员会办公室

主任委员签字	徐[illegible]	日期	2017.1.4
